# Supplementary material for: Federated Learning for multi-omics: a performance evaluation in Parkinson’s disease
Source: bioRxiv. 2023 Oct 6:2023.10.04.560604. Preprint. [Version 1] doi: 10.1101/2023.10.04.560604 (PMC10659429; doi:10.1101/2023.10.04.560604)
Supplement: Supplementary file 1 [file media-2.docx]

| **Algorithm Name** | **Central Learner API** | **Federated Local Learner API** | **Federated Weight Aggregation Method** | **Federated Learning API** |
| --- | --- | --- | --- | --- |
| Logistic Regression | Scikit Learn | Scikit Learn | FedAvg, FedProx | Flower Framework |
| MLP Classifier | Scikit Learn | Scikit Learn | FedAvg, FedProx | Flower Framework |
| SGD | Scikit Learn | Scikit Learn | FedAvg | NVIDIA Flare |
| RF XGBoost Classifier | DMLC | DMLC | FedAvg | NVIDIA Flare |

**Supplementary Table 1:** The description of frameworks used to implement central, and federated learning models.
