# Supplementary material for: Federated Learning for multi-omics: a performance evaluation in Parkinson’s disease
Source: bioRxiv. 2023 Oct 6:2023.10.04.560604. Preprint. [Version 1] doi: 10.1101/2023.10.04.560604 (PMC10659429; doi:10.1101/2023.10.04.560604)
Supplement: Supplementary file 2 [file media-3.docx]

| **Class Name** | **Algorithm Package** | **Package Version** |
| --- | --- | --- |
| LogisticRegression | sklearn | 1.3.0 |
| RandomForestClassifier | sklearn | 1.3.0 |
| AdaBoostClassifier | sklearn | 1.3.0 |
| GradentBoostingClassifier | sklearn | 1.3.0 |
| SGD | sklearn | 1.3.0 |
| SVC | sklearn | 1.3.0 |
| MLPClassifier | sklearn | 1.3.0 |
| KNNClassifier | sklearn | 1.3.0 |
| LinearDiscriminantAnalysis | sklearn | 1.3.0 |
| BaggingClassifier | sklearn | 1.3.0 |
| XGBClassifier | xgboost | 1.7.6 |
| XGBRFClassifier | xgboost | 1.7.6 |

**Supplementary Table 2:** The class names, algorithm packages, and package versions used to implement local learners in federated models, and central machine learning models.
