# Supplementary material for: Federated Learning for multi-omics: a performance evaluation in Parkinson’s disease
Source: bioRxiv. 2023 Oct 6:2023.10.04.560604. Preprint. [Version 1] doi: 10.1101/2023.10.04.560604 (PMC10659429; doi:10.1101/2023.10.04.560604)
Supplement: Supplementary file 1 [file media-1.pdf]

Supplementary Information

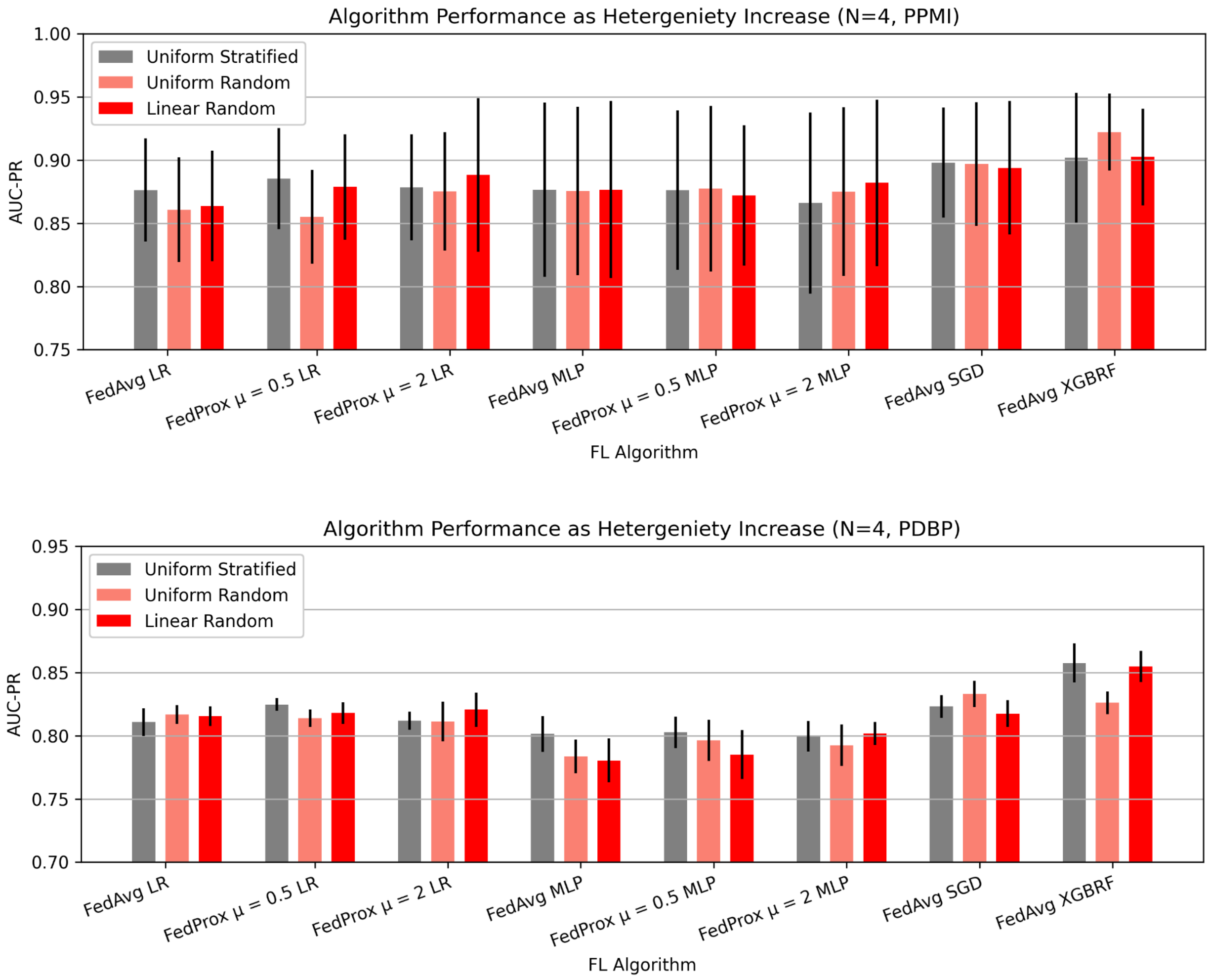

Supplementary Figure 1: The AUC-PR for a federation of 4 clients, for several split methods.

Uniform stratified sampling, representing the most homogenous data distribution method, while uniform random, and linear random represent increasingly heterogeneous client distributions. Presented data is mean score and standard deviation resulting from cross validation.

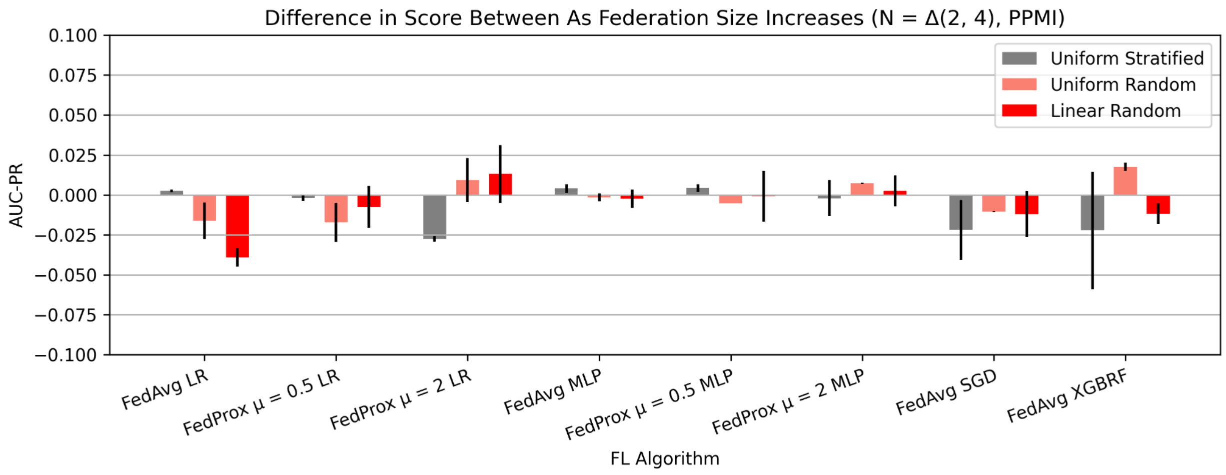

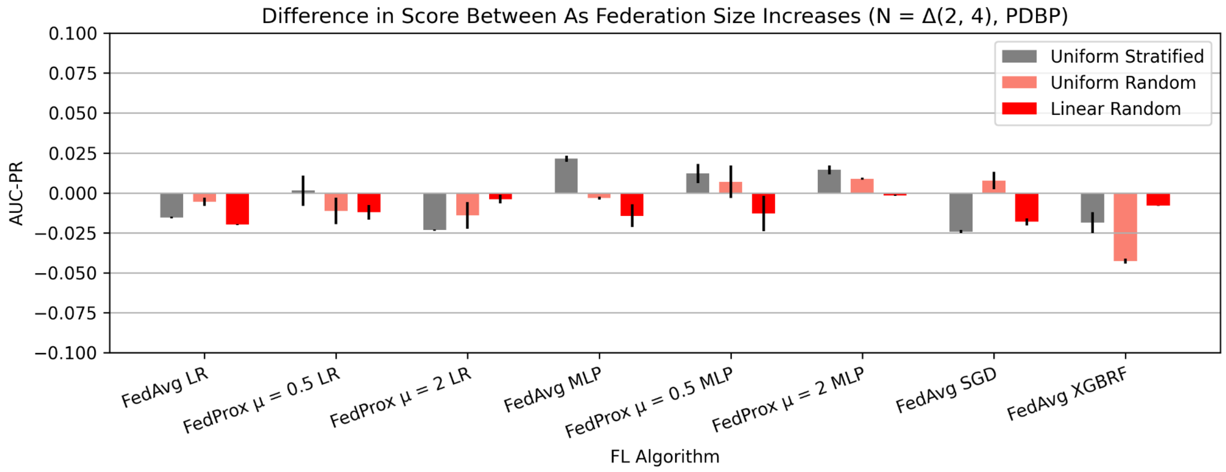

Supplementary Figure 2: The difference between AUC-PR scores between 2 and 4 clients, for several split methods.

Uniform stratified sampling, representing the most homogenous data distribution method, while uniform random, and linear random represent increasingly heterogeneous client distributions. Presented data is mean score and standard deviation resulting from cross validation.
